# Virtual Patient Simulations Using Social Robotics Combined With Large Language Models for Clinical Reasoning Training in Medical Education: Mixed Methods Study

**DOI:** 10.2196/63312

**Published:** 2025-03-03

**Authors:** Alexander Borg, Carina Georg, Benjamin Jobs, Viking Huss, Kristin Waldenlind, Mini Ruiz, Samuel Edelbring, Gabriel Skantze, Ioannis Parodis

**Affiliations:** 1 Division of Rheumatology Department of Medicine Solna Karolinska Institutet, Karolinska University Hospital, and Center for Molecular Medicine (CMM) Stockholm Sweden; 2 Department of Neurobiology, Care Sciences and Society Karolinska Institutet Stockholm Sweden; 3 Division of Clinical Epidemiology Department of Medicine Solna Karolinska Institutet, Karolinska University Hospital Stockholm Sweden; 4 Department of Clinical Science, Intervention and Technology Karolinska Institutet Stockholm Sweden; 5 School of Health Sciences Örebro University Örebro Sweden; 6 School of Education, Culture and Communication Mälardalen University Västerås Sweden; 7 Division of Speech Music and Hearing Royal Institute of Technology Stockholm Sweden; 8 Department of Rheumatology Faculty of Medicine and Health Örebro University and Örebro University Hospital Örebro Sweden

**Keywords:** virtual patients, clinical reasoning, large language models, social robotics, medical education, sustainable learning, medical students

## Abstract

**Background:**

Virtual patients (VPs) are computer-based simulations of clinical scenarios used in health professions education to address various learning outcomes, including clinical reasoning (CR). CR is a crucial skill for health care practitioners, and its inadequacy can compromise patient safety. Recent advancements in large language models (LLMs) and social robots have introduced new possibilities for enhancing VP interactivity and realism. However, their application in VP simulations has been limited, and no studies have investigated the effectiveness of combining LLMs with social robots for CR training.

**Objective:**

The aim of the study is to explore the potential added value of a social robotic VP platform combined with an LLM compared to a conventional computer-based VP modality for CR training of medical students.

**Methods:**

A Swedish explorative proof-of-concept study was conducted between May and July 2023, combining quantitative and qualitative methodology. In total, 15 medical students from Karolinska Institutet and an international exchange program completed a VP case in a social robotic platform and a computer-based semilinear platform. Students’ self-perceived VP experience focusing on CR training was assessed using a previously developed index, and paired 2-tailed *t* test was used to compare mean scores (scales from 1 to 5) between the platforms. Moreover, in-depth interviews were conducted with 8 medical students.

**Results:**

The social robotic platform was perceived as more authentic (mean 4.5, SD 0.7 vs mean 3.9, SD 0.5; odds ratio [OR] 2.9, 95% CI 0.0-1.0; *P*=.04) and provided a beneficial overall learning effect (mean 4.4, SD 0.6 versus mean 4.1, SD 0.6; OR 3.7, 95% CI 0.1-0.5; *P*=.01) compared with the computer-based platform. Qualitative analysis revealed 4 themes, wherein students experienced the social robot as superior to the computer-based platform in training CR, communication, and emotional skills. Limitations related to technical and user-related aspects were identified, and suggestions for improvements included enhanced facial expressions and VP cases simulating multiple personalities.

**Conclusions:**

A social robotic platform enhanced by an LLM may provide an authentic and engaging learning experience for medical students in the context of VP simulations for training CR. Beyond its limitations, several aspects of potential improvement were identified for the social robotic platform, lending promise for this technology as a means toward the attainment of learning outcomes within medical education curricula.

## Introduction

Virtual patients (VPs) are computed interactive simulations of clinical scenarios for educational or learning purposes [1]. These tools are used to address several learning outcomes in health professions education (HPE) curricula [2]. VP platforms can be designed in a linear fashion, where medical information and treatment options are gathered in a fixed order without consequences, or with a branched design, where interactions also impact the outcome of the case [3]. VPs have been proposed as an effective educational tool for practicing clinical reasoning (CR) in undergraduate medical education [4] while also ensuring that medical students encounter representative patient cases, which might not always be possible to encounter during clinical rotations [5-7]. CR is an essential skill for health care practitioners, and its inadequacy may threaten patient safety, making it a key goal in HPE [8-10]. An essential aspect of VP simulation is the interface, which allows the user to interact with the VP in multiple ways. A common feature of VP platforms is that they can facilitate the obtainment of medical history, the conduct of physical examinations, and referral for laboratory tests and the provision of their findings to culminate in a reasonable diagnosis and management plan [11].

Previous research shows that medical students perceive VP simulation as a satisfactory educational activity for training CR while they acknowledge the lack of complexity and level of interactivity compared with authentic patient encounters [12]. Interestingly, students have been shown to perform better at examinations after exposure to VP simulations compared to other educational activities with similar learning outcomes, but the positive effect of VPs on CR training is still unclear [2,13].

Recent technological developments have enabled the integration of dialogue systems with natural language processing capabilities, embodied conversational agents [14], and social robots [15] to enhance the interactivity and realism of VP interactions. The emergence of large language models (LLMs) and their application in dialogue systems and social robots have introduced novel possibilities within this field [16,17]. However, their use for VPs has been limited to preliminary endeavors [18-21]. To the best of our knowledge, no prior studies have used LLMs in conjunction with a social robot for VP simulation and evaluated their effectiveness in facilitating CR training.

We hypothesized that the integration of an LLM in a social robotic modality for the presentation of VPs would involve several advantages. More specifically, we hypothesized that the physical presence of a social robot, combined with its nonverbal communication capabilities, would enhance engagement and immersion in the learning experience. This integration of visual and social cues, along with advanced verbal interactions, could facilitate a more comprehensive information-gathering process during the VP encounter until a diagnosis has been made and a management plan has been proposed compared with using only LLMs for VP presentation or conventional computer-based platforms.

While VPs can be valuable across many medical disciplines, this study focuses on their application in rheumatology education. Rheumatic diseases affect a substantial proportion of the population and are considered a significant public health concern [22-24]. These conditions often present complex diagnostic challenges that make them particularly suitable for CR training through VP simulations. To ensure that patients with rheumatic diseases receive appropriate care, it is crucial to provide evidence-based training in CR for medical students [25]. The aim of this study was to explore the potential added value of and potential limitations with a social robot combined with an LLM in comparison to a conventional, computer-based, semilinear VP platform presenting rheumatic conditions for medical students’ CR training.

## Methods

An interventional, explorative, proof-of-concept study was conducted between May and July 2023. We combined quantitative and qualitative methodology to assess medical students’ self-perceived CR training using a previously developed index for VP design evaluation [26] as well as in-depth interviews.

### Participants

In total, 15 medical students from Karolinska Institutet (KI) were recruited in the study either from a 5-week elective course for third-year students or a 4-week exchange program for international students, both pertaining to inflammation within internal medicine, including rheumatology, gastroenterology, dermatology, nephrology, and infectious diseases. All students had completed the sixth term of their medical education. More specifically, prior to the course, students from KI had completed 2 full years of preclinical medical education (eg, anatomy, physiology, and pathophysiology) and 1 year of clinical rotations in internal medicine. International students had also completed the preclinical phase of their medical education and had varying clinical backgrounds, with at least 1 full year of clinical rotations in internal medicine. While all students had prior experience with patient encounters and were fluent in English, none were native English speakers, and their previous experience of communicating with patients in English varied. In total, 8 students also agreed to participate in in-depth interviews, scrutinizing their experience with the social robotic platform compared with the conventional computer-based VP platform with regard to CR training.

### Ethical Considerations

The study protocol was reviewed and approved by the Swedish Ethical Review Authority (registration 2022-04437-01). Written informed consent was obtained from all study participants prior to enrollment. Participants had the ability to opt out at any time. All data were deidentified prior to analysis. No compensation was provided for participation in this study.

### Development and Implementation of VP Cases

A VP case illustrating a common rheumatic disease (ie, polymyalgia rheumatica) was developed for presentation in a social robotic platform from Furhat robotics [27] and a conventional semilinear computer-based platform (ie, the virtual interactive case simulator [VIC]) [28]. The case development in both platforms adhered to distinct principles for VP case development proposed by Posel et al [29].

First, we identified the content of the VP case and selected the design. Between a linear and an explorative design, we anticipated advantages in using an explorative design, as it more closely resembles real-life clinical situations. We then organized the VP content by clarifying the purpose of each step within the VP cases. Next, we tailored the delivery of the VP case by adjusting the level of difficulty to align with the medical students’ prior knowledge of rheumatic diseases. The VP cases were continuously evaluated through feedback from students and peers before, during, and after development. Integrity and confidentiality were maintained by creating typical case scenarios without using any information from real patients.

The students were tasked to perform the VP case individually in both platforms to facilitate paired comparisons. The case was considered within a particular situational context to be a primary care encounter. The students were assigned the role of an attending physician. The case was completed when the students had obtained sufficient information to make a diagnosis and suggest a management plan for the patient.

After interaction with each VP platform, students immediately filled out a questionnaire evaluating the VP design with an emphasis on CR training and provided optional free-text evaluation within seven themes: (1) authenticity of patient encounter, (2) professional approach in the consultation, (3) coaching during consultation, (4) learning effect of consultation, (5) overall judgment of case work-up, (6) special strengths of the case, and (7) special weaknesses of the case [26]. The questionnaire consisted of 14 questions, of which the first 12 questions (corresponding to themes 1 to 5) were graded using a 5-point scale, where 1=strong disagreement and 5=strong agreement, whereas responses to the last 2 questions (corresponding to themes 6 and 7) were only provided in the form of free text. This questionnaire was specifically developed for evaluating the design of VP platforms with an emphasis on CR training. Its development combined a theoretical framework for CR training along with the involvement of an international team of VP experts, supporting its content validity [26]. The questionnaire is provided in Figure S1 in Multimedia Appendix 1.

### Social Robotic Platform

The social robot serving as the embodiment for the novel VP platform features an animated face back-projected onto a translucent mask and a neck with 3 degrees of freedom, allowing for natural head movements. The social robot consists of a built-in speaker to convey dialogue and a microphone to perceive spoken phrases that are later interpreted using a speech-to-text synthesis. Moreover, the social robot uses computer vision to track multiple users in real time, performing facial expression analysis, head pose estimation, and user distance measurement. It includes a face detection system, multiuser tracking, depth estimation, spatial modeling, and a deep neural network–based face recognition for tracking users over time [27]. The animated face expresses subtle emotions through facial expressions and gaze behavior, crucial for conveying a patient’s internal state [30].

The platform combines the robot-steering software development kit FurhatSDK [27] and the LLM OpenAI’s GPT-3.5-turbo chat completion model [31]. To maintain authenticity and avoid responses sounding like an assistant, we used a single user message containing the required information to generate a VP response. This prompt included a patient case description, the last 10 dialogue turns, and instructions to generate the next dialogue line. A shortened example of a prompt is provided in Figure S2 in Multimedia Appendix 1.

We also used the LLM to generate facial expressions, essential for reflecting the patient’s emotional states during interactions, similar to the methods described by Irfan et al [16]. At the beginning of each utterance, anchor points were inserted at phrase boundaries within the text. The LLM was next prompted to incorporate suitable facial expressions at each anchor point, selecting from the FurhatSDK’s available expressions, for example, sad, happy, or surprised facial expressions, illustrating a variety of nonverbal expressions adapted to the context of the conversation.

The LLM calls for generating dialogue responses and facial expressions were separate. First, the dialogue response was generated, and the response was translated using a text-to-speech synthesis. While the response was being synthesized, a second call was made to the LLM to generate the list of facial expressions related to each anchor point. While the robot was speaking, the system kept track of when each word had been spoken, so that the relevant facial expression could be projected.

Using LLMs can cause response delays, complicating turn-taking, as users might not realize that the robot is generating a reply [16]. This issue is worsened when there is no provision for barge-in. To address this, various approaches can be adopted, such as the use of a turn-holding cue to indicate that the robot is actively “thinking” about a response [32]. We adopted a gaze aversion technique [33] combined with LED lights at the base of the robot to communicate its turn-taking status, indicating whether it is speaking or listening.

### Virtual Interactive Case Simulator

In VIC, a semilinear approach is used, ensuring a consistent beginning and end for each case while allowing users to explore the case environment in their preferred order [3]. Every case starts with a brief patient summary and finishes with a diagnostic verdict and management approach, presented in a multiple-choice structure. This interface allows the user to investigate, for example, the patient’s medical history, administer a physical examination, and retrieve laboratory test results. Interaction with this platform is facilitated entirely using prewritten clickable options, with no reliance on natural language processing methodologies.

### Interviews

We carried out one-on-one, semistructured interviews with medical students following the completion of the VP case sessions using both platforms. This was performed on the same day, immediately after having completed the VP cases in both platforms. We developed an interview guide that consisted of questions concerning the participants’ experiences with the VP platforms as tools for learning and CR training as well as their views on potential applications and integration of these platforms within medical curricula. The interview guide is provided in Figure S3 in Multimedia Appendix 1.

Interviews were held at Karolinska University Hospital during clinical placements as described earlier and lasted from 20 to 30 minutes each. The interview data were stored in dedicated spaces at the Department of Medicine Solna, KI. The recorded audio files were transcribed verbatim, and the transcripts were pseudonymized, ensuring the removal of any sensitive or personal identifiable information.

### Analysis

To compare responses to the quantitative part of the questionnaire (themes 1 to 5) relating to the robotic platform versus VIC, the paired 2-tailed *t* test was used to compare mean scores between the platforms. This analysis included both separate questions and summary scores within themes. Results from paired 2-tailed *t* tests are presented as the mean score (SD), 95% CI, odds ratio (OR), and *P* value. The statistical analysis was performed using the R statistical software (version 4.2.1; R Foundation for Statistical Computing). Differences generating *P* values <.05 were considered statistically significant.

Interview data were analyzed systematically for the identification of key themes. The thematic analysis was carried out following the approach described by Braun and Clarke [34]. First, the verbatim transcripts of the interviews were reviewed multiple times to ensure familiarity with the content. The interviews were analyzed individually as a first step, and then horizontally, that is, across interviews. International recommendations for integrating CR training into HPE curricula [10] were used as a guideline when coding words and phrases related to CR training. This coding process was iterative, with the researchers collaborating to identify, interpret, and refine the codes until the final themes emerged. The initial stages of the analysis and theme development were undertaken by 2 investigators (AB and BJ), while a third investigator (IP) supervised the process and contributed to the final selection of the themes.

All authors contributed to the interpretation of the results, bringing diverse perspectives to the analysis. AB was at the time of writing a medical intern and PhD student within medical pedagogy, CG was a nurse specialized in intensive care, BJ was a medical student during the fourth year of the KI medical program, VH was a resident physician in rheumatology, KW was a consultant rheumatologist, MR was a consultant endocrinologist, SE was a senior lecturer and associate professor in medical pedagogy, GS was a professor in speech technology, one of the founders of the social robot used in the project, and chief scientist at Furhat Robotics. IP was a senior consultant rheumatologist, associate professor of rheumatology, and educator within the KI medical program. This multidisciplinary team contributed complemental approaches and views during different stages of the analysis, substantially enhancing the overall interpretation of the data.

## Results

### Questionnaire

In total, 6 (40%) of the 15 medical students were women, and 9 (60%) were men. A total of 11 (73%) students declared no previous experience with VP platforms. The mean age of the students was 23.9 (SD 4.8) years. Response frequencies to all quantitative themes (1 to 5) are provided in Tables S1-S5 in Multimedia Appendix 2.

**Figure 1:**
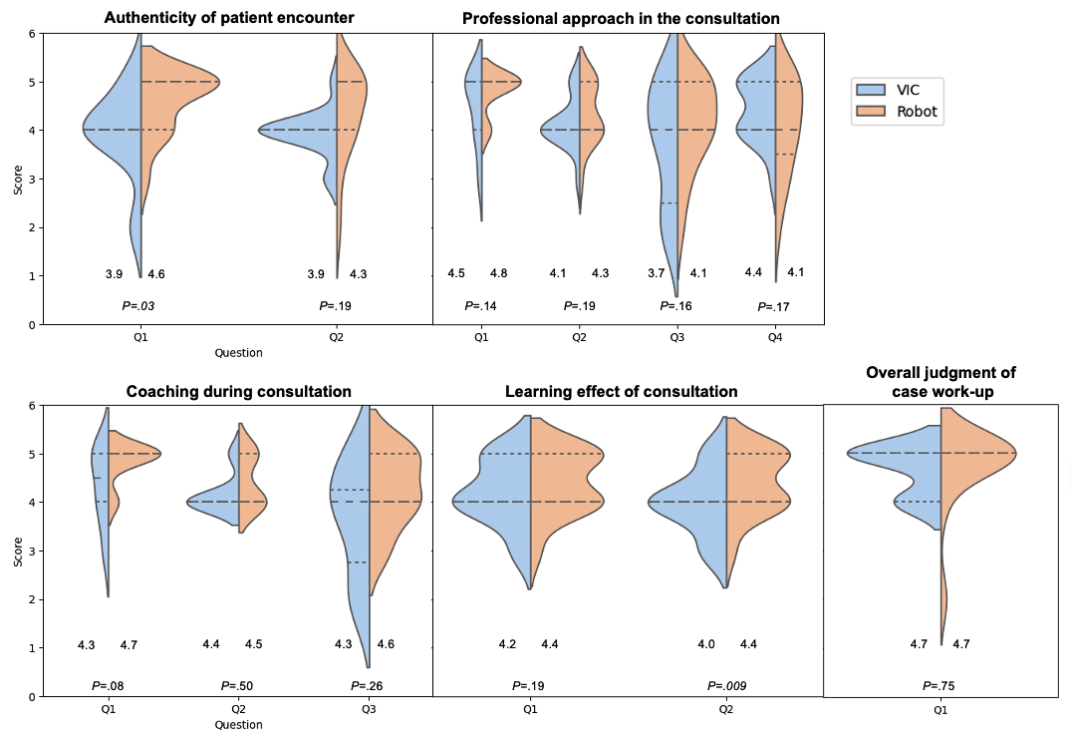
Violin plots illustrating distributions of scores from students’ responses to questions within quantitative themes (1 to 5) with regard to the social robotic platform versus VIC. Lines within violin plots denote median values and IQRs. Numbers at the bottom of the plots refer to mean values. Statistically significant *P* values are in italics format. Q: question; VIC: virtual interactive case simulator.

The students perceived the social robotic platform as more authentic compared with VIC with regard to decision-making (mean 4.4, SD 0.6 vs mean 3.9, SD 0.9; OR 3.2, 95% CI 0.1-1.2; *P*=.03) as well as beneficial regarding the preparation of taking care of a real-life patient with the same complaint (mean 4.4, SD 0.6 vs mean 4.0, SD 0.7; OR 4.2, 95% CI 0.1-0.7; *P*=.009). We documented a numerical difference in favor of the social robotic platform with regard to the appropriateness of the VP case’s level of difficulty (mean 4.7, SD 0.5 vs mean 4.3, SD 1.0; OR 2.4, 95% CI –0.1 to 0.9; *P*=.08); however, this difference did not reach statistical significance. Detailed results are shown in Table 1 and Figure 1.

**Table 1:** Comparison of mean scores using paired 2-tailed *t* test within each question of the clinical reasoning questionnaire (N=15).

Theme and variable			Social robot, mean (SD)		VIC^a^, mean (SD)		OR^b^ (95% CI)		*P* value
**Authenticity of patient encounter**									
	Question 1	4.6 (0.6)		3.9 (0.9)		3.2 (0.1 to 1.2)		*.03^c^*	
	Question 2	4.3 (0.9)		3.9 (0.5)		1.9 (–0.2 to 1.0)		.19	
**Professional approach in the consultation**									
	Question 1	4.8 (0.4)		4.5 (0.7)		2.1 (–0.1 to 0.8)		.14	
	Question 2	4.3 (0.6)		4.1 (0.5)		1.9 (–0.1 to 0.5)		.19	
	Question 3	4.1 (0.9)		3.7 (1.2)		2.0 (–0.2 to 1.0)		.16	
	Question 4	4.1 (1.0)		4.4 (0.6)		0.5 (–0.8 to 0.2)		.17	
**Coaching during consultation**									
	Question 1	4.7 (0.5)		4.3 (1.0)		2.4 (–0.1 to 0.9)		.08	
	Question 2	4.5 (0.5)^d^		4.4 (0.7)^d^		1.4 (–0.3 to 0.6)		.50	
	Question 3	4.6 (0.5)^d^		4.3 (0.9)^d^		1.8 (–0.3 to 0.9)		.26	
**Learning effect of consultation**									
	Question 1	4.4 (0.6)		4.2 (0.7)		1.9 (–0.1 to 0.5)		.19	
	Question 2	4.4 (0.6)		4.0 (0.7)		4.2 (0.1 to 0.7)		*.009*	
**Overall judgment of case work-up**									
	Question 1	4.7 (0.8)		4.7 (0.5)		1.2 (–0.4 to 0.5)		.75	

^a^VIC: virtual interactive case simulator.

^b^OR: odds ratio.

^c^Statistically significant *P* values are in italics format.

^d^n=13.

**Figure 2:**
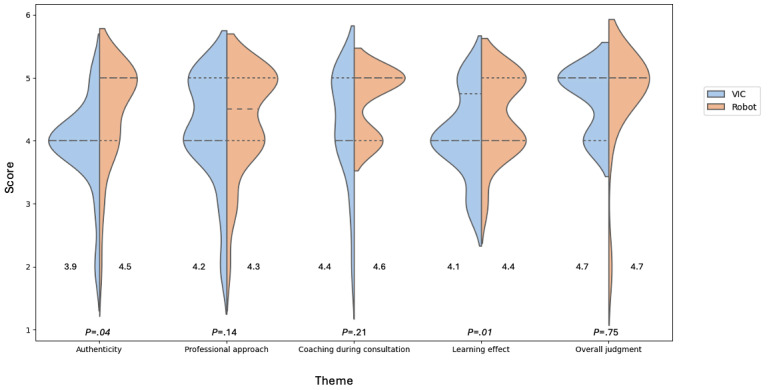
Violin plots illustrating distributions of summary theme scores of quantitative themes (1 to 5) in the social robotic platform versus VIC. Lines within violin plots denote median values and IQRs. Numbers at the bottom of the plots denote mean values. Statistically significant *P* values are in italics format. VIC: virtual interactive case simulator.

Summary scores within themes demonstrated superiority of the social robotic platform compared with VIC both regarding authenticity (mean 4.5, SD 0.7 vs mean 3.9, SD 0.5; OR 2.9, 95% CI 0.0-1.0; *P*=.04) and learning effect of the consultation by feeling more prepared to care for a real-life patient with a similar complaint (mean 4.4, SD 0.6 vs mean 4.1, SD 0.6; OR 3.7, 95% CI 0.1-0.5; *P*=.01), as illustrated in Figure 2 and Table 2.

Open-ended themes (6 and 7) highlighted the special strengths and limitations with both platforms. Students perceived the social robotic platform as a good way to simulating realistic patient conversations and providing an interactive and engaging experience. Limitations of the social robot included the lack of laboratory results and the lack of modality for training physical examination, as well as that conversations sometimes felt mechanical due to their turn-based structure and due to the fact that students were interrupted by the robot if their verbal pause was too long since this pause was perceived as the end of turn.

By contrast, students perceived the VPs presented through VIC as a good tool for structured learning and revision of the case. VIC provided useful feedback and information regarding various clinical investigations. However, the fixed question format in VIC was a limiting factor, as was the lack of the possibility to ask the VP follow-up questions. Some students noted that VIC did not provide a feeling that resembled a real patient encounter to the same extent as the social robot. Detailed responses are shown in Tables S6 and S7 in Multimedia Appendix 2.

**Table 2:** Comparisons of summary theme scores from the clinical reasoning questionnaire between the social robotic platform and VIC^a^ (N=15).

Theme	Social robot, mean (SD)	VIC, mean (SD)	OR^b^ (95% CI)	*P* value
Authenticity of patient encounter	4.5 (0.7)	3.9 (0.5)	2.9 (0.0 to 1.0)	*.04*
Professional approach in the consultation	4.3 (0.5)	4.2 (0.5)	2.0 (–0.1 to 0.4)	.16
Coaching during consultation	4.6 (0.4)^c^	4.4 (0.6)^c^	1.5 (–0.2 to 0.7)	.21
Learning effect of consultation	4.4 (0.6)	4.1 (0.6)	3.7 (0.1 to 0.5)	*.01^d^*
Overall judgment of case work-up	4.7 (0.8)	4.7 (0.5)	1.2 (–0.4 to 0.5)	.75

^a^VIC: virtual interactive case simulator.

^b^OR: odds ratio.

^c^n=12.

^d^Statistically significant *P* values are in italics format.

### Interviews

#### Overview

A total of 8 medical students participated in in-depth interviews. In total, 5 (63%) students were women, and 3 (37%) students were men. The students’ mean age was 21.5 (SD 1.9) years. Thematic analysis resulted in four themes: (1) realism and immersion, (2) skill acquisition and development, (3) procedural limitations, and (4) potential for improvement (Table S8 in Multimedia Appendix 2).

#### Realism and Immersion

The theme “realism and immersion” was divided into two subthemes: (1) authenticity and (2) interactivity and engagement. Students perceived the experience with the LLM-empowered robotic platform as a realistic and authentic patient encounter, outperforming the computer-based platform in these aspects. Within the subtheme “authenticity,” students described that the realistic impression conferred from the social robotic platform made them feel like being the treating physician in a real-life scenario, with responsibilities toward the presented patient. Relevant quotes are presented in Textbox 1.

**Relevant quotes by theme “realism and immersion” and subthemes from in-depth interviews.**

**Subtheme: authenticity**

- “It felt close, like if I were with a real patient” [Male student, 22 years].
- “[...] you are asking the questions and in real time there is a response, and it is like a real conversation. [...] I definitely felt like the treating physician with the robot, knowing that it is up to me to ask the questions and to take in what the patient is saying” [Female student, 22 years].
- “Because the patient was expressing how he was in pain, and how, you know, the pain was bothering him. [...] So yeah, it felt quite intimate at that time. And it felt like I was a physician taking care of a patient” [Male student, 25 years].

**Subtheme: interactivity and engagement**

- “The robot is more interactive compared to the computer. The computer [...] is of course very helpful. But with the robot, it is more fun because you see this robot talking to you and answering your questions in real time” [Female student, 20 years].
- “[...] the computer platform is more passive. You don’t have to be so productive” [Male student, 25 years].
- “I think the computer-clicking, the computer platform, is less demanding. I would say it is on a lower level of interactivity than the robot” [Male student, 22 years].

The social robotic platform facilitated a highly interactive and engaging experience, which was described to be superior to VIC. Within the subtheme “interactivity and engagement,” students expressed that the robot required active participation, which was experienced as more engaging and immersive than passively clicking through the computer-based case. The face projection of the robot added to the feeling of interactivity (Textbox 1).

#### Skill Acquisition and Development

The theme “skill acquisition and development” consisted of three subthemes: (1) CR skills, (2) communication skills, and (3) emotional skills. Within the subtheme “CR skills,” students perceived the robot as an effective tool for acquirement of CR skills by engaging in an active dialogue, practicing the obtainment of medical history, reasoning across different explorative approaches, and planning for various treatment strategies. Students believed that this was due to them actively having to think about the patient’s symptoms and ask relevant questions based on the responses they were given. They acknowledged that the computer-based case was more informative, but in the latter, they lacked the ability to ask follow-up questions, which was possible with the LLM-empowered social robot (Textbox 2).

Students experienced that the robotic platform helped them practice “communication skills,” especially by facilitating active communication, engaging them in thinking about which questions to ask and how to phrase them in real time (Textbox 2). While the social robotic platform allowed students to feel closer to VPs compared with the computer-based platform, students perceived that the practice of “emotional skills,” such as empathy, was inferior with both platforms compared to real-life patient encounters (Textbox 2).

**Relevant quotes by theme “skill acquisition and development” and subthemes from in-depth interviews.**

**Subtheme: clinical reasoning skills**

- “To direct your questions. Patients often come with something very diffuse, like pain. And then you have to think, ‘pain, duration, onset [...]’ to quickly orient yourself in the patient’s problem and then direct your questions afterwards. It’s a great tool for practicing that” [Male student, 25 years].
- “The computer case, I would say, is very comprehensive. It gave us the data on pretty much everything, the lab tests, the patient’s history. It has data on everything” [Female student, 20 years].
- “I remember asking ‘does it get better with a certain medication?’ or ‘how long does it last?,’ ‘when was the last time you had it?’ [...] And I felt like that’s something I couldn’t have found in the computer-based case. It didn’t give me that information” [Female student, 21 years].

**Subtheme: communication skills**

- “With the robot, I felt I got to use my skills of how to speak to a patient. [...] you really have to use your medical knowledge to ask [the right] questions and get answers” [Female student, 21 years].

**Subtheme: emotional skills**

- “Because you have to not only ask about the medical stuff, but also you have to have a dialogue [...] as you do with a human being. So, yeah, you feel closer [to the patient] with the robot compared with the computer” [Female student, 22 years].
- “[...] Just like having that [...] empathy or when you’re just talking to a human, looking them into the eyes and saying, ‘oh, I’m very sorry for you that you have had these headaches.’ You don’t really see the pain in someone through a robot” [Female student, 21 years].
- “Maybe what it lacked is [...] emotions. Not like patients whom you need to reassure” [Female student, 19 years].

#### Procedural Limitations

The theme “procedural limitations” consisted of two subthemes: (1) technical limitations and (2) user-related challenges. Within the subtheme “technical limitations,” students expressed technical problems such as being interrupted by the robot during conversational pauses that were conceived as the end of a spoken sentence. This resulted in the perception of a time constraint while responding to the platform, which sometimes hindered the flow of the interaction (Textbox 3). The subtheme “user-related challenges” sheds light on challenges relating to language barriers as well as a need for adapting to the conversational style of the social robot (Textbox 3).

**Relevant quotes by theme “procedural limitations” and subthemes from in-depth interviews.**

**Subtheme: technical limitations**

- “Well, you have a time when you can talk and another time when you can’t talk. Apart from the clinical reasoning, you have to pay attention to when [you should talk]. It can be disturbing” [Male student, 22 years].
- “[...] you have to complete your sentence, or else it might cut you off in the middle and start giving you responses” [Female student, 22 years].

**Subtheme: user-related challenges**

- “It was a bit difficult. Partly because you’re not used to speaking English with patients. But also because of how [the robot’s] artificial pauses could disturb” [Female student, 23 years].
- “I guess for some people, who are limited by [...] language barriers in English, it might be difficult for them” [Female student, 20 years].

#### Potential for Improvement

Within the theme “potential for improvement,” students expressed how specific technological aspects could be improved in the social robotic platform. Students envisioned the potential of more realistic facial expressions and a multitude of patient personalities, which both could enhance the degree of interactivity and perception of realism, presumably contributing to increased engagement and increased chance of achieving the intended learning outcomes. This potential was limited to the social robotic platform (Textbox 4).

**Relevant quotes by theme “potential for improvement” from in-depth interviews.**

- “Maybe [improvements] on the face. To work with the voice. And to broaden the responses. So that it might be more complex” [Male student, 20 years].
- “Like in real life, all patients won’t give you the same amount of information. So, I think it can be interesting, apart from [presenting] different diseases, also present different types of personalities” [Male student, 22 years].
- “[...] various personalities and patients who are not cooperative or might need many questions to receive certain responses need different ways of being handled; one patient is not another alike. I think you have to adapt to whichever type of patient you have in front of you” [Female student, 21 years].

## Discussion

### Principal Findings

We developed a novel social robotic VP platform in conjunction with an LLM and present herein results from a proof-of-concept study that explored the self-perceived usefulness and effectiveness of this new pedagogical modality for CR training of medical students compared with a conventional, computer-based, semilinear VP platform. The study included a quantitative comparison between the 2 platforms and qualitative in-depth interviews. Results indicated that VP exercise with the social robotic platform was perceived as more authentic and immersive as well as conferring a self-perceived benefit regarding the learning effect compared with the computer-based platform. Overall, students experienced that the social robot could promote training of CR, communication, and emotional skills. Students highlighted the limitations of the social robot relating to technical and user-related aspects and provided suggestions for potential improvements within the context of CR training.

Medical students perceived the social robotic platform as more authentic compared to the computer-based platform, as demonstrated by significantly higher scores in the authenticity theme of the questionnaire. The qualitative analysis of the interviews further supported these findings, with students describing the social robotic platform as providing a realistic and immersive experience that closely resembled a real patient encounter. These results are consistent with previous studies that highlight the role of interactivity and complexity within VP platforms in enhancing learning toward defined outcomes [3,12,35]. It was based on this premise that we designed and developed the social robotic VP platform for enhanced interactivity, integrating an LLM for enhanced complexity.

The quantitative analysis revealed that students perceived the social robotic platform as preferable to the computer-based platform in terms of the learning effect of the consultation, particularly regarding the perceived transferability of skills to real-life clinical scenarios. The qualitative analysis of the interviews provided further insights into the specific skills that students believed they acquired through interaction with the robotic platform. Students reported that the robotic platform facilitated the development of CR skills by engaging them in active dialogue, requiring them to think critically about the patient’s symptoms, and allowing them to ask relevant follow-up questions. Additionally, students experienced that the robotic platform helped them practice communication skills by encouraging them to actively engage in conversation and think about how to phrase their questions, which has previously been highlighted as an important aspect for CR training using VPs [29].

The qualitative analysis of the interviews revealed several limitations and challenges associated with the social robotic platform. Students reported technical limitations, such as being interrupted by the robot during conversational pauses, which sometimes made the conversation feel mechanical and hindered the flow of the interaction. This could negatively affect the perceived authenticity of the social robotic platform, highlighting the need for technical improvements in this area as a priority for future development.

Students also reported the benefits of the computer-based platform compared with the social robot, noting it as more informative. Another advantage, though not directly compared in this study, is that the computer-based platform VIC is more accessible, as students can access these cases on their personal computers, whereas the social robot is only available when students are physically present with the modality.

User-related challenges, such as language barriers and the need to adapt to the conversational style of the robot, were also identified. These findings highlight the importance of addressing technical limitations and providing adequate support to users to ensure a smooth and effective learning experience. Despite these limitations, students also expressed that the social robot introduced potential for additional improvements that cannot be facilitated by a conventional computer-based platform, such as more realistic facial expressions and the incorporation of various patient personalities, which can be anticipated to further enhance interactivity and realism.

### Limitations and Strengths

This study has several limitations. First, the sample size was small, which limits the generalizability of the findings. Second, the study was conducted in a single center, and the results may not be representative of the experience of medical students in other settings. Third, CR is a complex skill, and how its training is facilitated cannot be comprehensively assessed by a single questionnaire, whose validation may not encompass all its aspects. The questionnaire used in this study was specifically designed to evaluate VP design with a particular focus on CR, but it did not directly assess the acquisition of CR skills; this aspect was instead explored in the qualitative part of the study through in-depth interviews. Our study also has several strengths. The use of a mixed quantitative and qualitative methodological approach allowed for a comprehensive evaluation of students’ perceptions and experiences with the VP platforms. Importantly, the study compared a novel social robotic platform in conjunction with an LLM, to our knowledge the first of its kind for medical pedagogy, with a conventional computer-based platform, providing valuable insights into the added value of social robots and artificial intelligence in VP simulations for CR training in HPE curricula.

### Implications

Overall, the social robotic VP platform shows promise for enhancing CR training by bridging theoretical learning and clinical practice. Its heightened authenticity and interactivity bring it closer to real-life patient interactions, enabling medical students to practice CR in a realistic environment before engaging with actual patients. However, further evaluation and comparisons with other LLM-driven modalities are needed to assess the unique benefits of using a physical robot versus an LLM alone for VP simulation. Given current accessibility limitations, the platform may be most effective as part of a hybrid approach, combining traditional computer-based VPs for broader access with scheduled sessions using social robotic VPs to deepen skill development. This approach could optimize educational impact while managing practical resource constraints across various medical education settings.

### Conclusions

This study demonstrates that a social robotic platform enhanced by an LLM could provide an authentic and engaging learning experience for medical students in the context of VP simulations for training CR. The social robotic platform was perceived as beneficial compared to a conventional computer-based platform in terms of authenticity and overall learning effect, and students reported that it facilitated training of CR, communication, and emotional skills. Despite the limitations and challenges identified, the potential for improvement in the social robotic platform lends promise for this technology in future medical education. Importantly, however, costs and logistics may constitute limitations for the implementation of this technology. Finally, it is worth noting that this was a proof-of-concept study, and further research with larger sample sizes and in diverse educational settings is warranted to fully understand the value of social robotic VPs in facilitating the attainment of learning outcomes within medical education curricula.

## Appendix

Multimedia Appendix 1 Supplementary figures, including the VP plattform evaluation questionnaire, an LLM prompt example, and the interview guide.

Multimedia Appendix 2 Supplementary tables, including responses to themes in the VP plattform evaluation questionnaire and identified themes derived from qualitative thematic analysis coding.

## Abbreviations

CR: clinical reasoning
HPE: health professions education
KI: Karolinska Institutet
LLM: large language model
OR: odds ratio
VIC: virtual interactive case simulator
VP: virtual patient

## Data Availability

The data generated or analyzed during this study can be provided by the corresponding author upon reasonable request.
