# Supplementary material for: Virtual Patient Simulations Using Social Robotics Combined With Large Language Models for Clinical Reasoning Training in Medical Education: Mixed Methods Study
Source: J Med Internet Res. 2025 Mar 3;27:e63312. doi: 10.2196/63312 (PMC11914843; doi:10.2196/63312)
Supplement: Multimedia Appendix 1 [file jmir_v27i1e63312_app1.pdf]

Figure S1. Questionnaire for the evaluation of medical students' perception of the design of VP platforms for CR training.

Please complete the survey below.

Thank you!

Student questionnaire concerning the learning and clinical reasoning experiences with virtual patients

About this questionnaire

This questionnaire is for students to evaluate their experiences with virtual patients, focusing on the development of clinical reasoning skills. The questionnaire is completely anonymous.

This questionnaire contains 14 items clustered into seven subsets. This instrument can be repeatedly administered to elicit student's experiences immediately following each workshop or 'play' of a virtual patient.

Please respond using the following 5-point scale:

1) Strongly disagree

2) Disagree

3) Neutral

4) Agree

5) Strongly agree

6) Not applicable

Please indicate briefly the reason(s) for your response for each question (optional).

**Participant information**	
Legal gender	◯ Male<br>◯ Female
Age	________________________<br>(Please provide your age as a number (For example: 27))
Which health profession education programme are you a student within?	◯ Medical programme<br>◯ Nursing programme<br>◯ Physiotherapy programme<br>◯ Occupational therapy programme

Which term of studies are you currently attending?	◯ 1<br>◯ 2<br>◯ 3<br>◯ 4<br>◯ 5<br>◯ 6<br>◯ 7<br>◯ 8<br>◯ 9<br>◯ 10<br>◯ 11<br>◯ 12
Do you have any previous experience of virtual patient simulations?	◯ Yes<br>◯ No
Please specify	________________________________
I performed the case	◯ Mikael<br>◯ Sara<br>◯ Rachel<br>◯ Simon<br>◯ Gordon
I completed the case	◯ By myself<br>◯ Together with another student<br>◯ In a group of students (more than 2)
Were your student colleague	◯ From the same profession<br>◯ From another profession<br>(Compared to the profession that you are a student within)
Were your student colleague	◯ A nursing student<br>◯ A physiotherapy student<br>◯ An occupational therapy student
Were your student colleagues	◯ From the same health profession<br>◯ From different health professions<br>(As compared with yourself)
Please specify the health professions that your student colleagues represented	__________________________<br>(For example: Student1: Medical programme, Student 2: Nursing programme, etc)
Date of case completion	__________________________<br>(YYYY-MM-DD)

Authenticity of patient encounter in the consultation	
While working on this case, I felt I had to make the same decisions a doctor would make in real life.	◯ Strongly disagree<br>◯ Disagree<br>◯ Neutral<br>◯ Agree<br>◯ Strongly agree<br>◯ Not applicable
Please indicate briefly the reason(s) for your response to the question above (optional)	________________________________
While working on this case, I felt I were the doctor caring for this patient	◯ Strongly disagree<br>◯ Disagree<br>◯ Neutral<br>◯ Agree<br>◯ Strongly agree<br>◯ Not applicable
Please indicate briefly the reason(s) for your response to the question above (optional)	________________________________

Professional approach in the consultation	
While working through this case, I was actively engaged in gathering the information (e.g., history questions, physical exams, lab tests) I needed, to characterise the patient's problem	◯ Strongly disagree<br>◯ Disagree<br>◯ Neutral<br>◯ Agree<br>◯ Strongly agree<br>◯ Not applicable
Please indicate briefly the reason(s) for your response to the question above (optional)	________________________________
While working through this case, I was actively engaged in revising my initial image of the patient's problem as new information became available	◯ Strongly disagree<br>◯ Disagree<br>◯ Neutral<br>◯ Agree<br>◯ Strongly agree<br>◯ Not applicable
Please indicate briefly the reason(s) for your response to the question above (optional)	________________________________
While working through this case, I was actively engaged in creating a short summary of the patient's problem using medical terms	◯ Strongly disagree<br>◯ Disagree<br>◯ Neutral<br>◯ Agree<br>◯ Strongly agree<br>◯ Not applicable
Please indicate briefly the reason(s) for your response to the question above (optional)	________________________________

While working through this case, I was actively engaged in thinking about which findings supported or refuted each diagnosis in my differential diagnosis	◯ Strongly disagree<br>◯ Disagree<br>◯ Neutral<br>◯ Agree<br>◯ Strongly agree<br>◯ Not applicable
Please indicate briefly the reason(s) for your response to the question above (optional)	________________________
**Coaching during consultation**	
I felt that the case was at the appropriate level of difficulty for my level of training	◯ Strongly disagree<br>◯ Disagree<br>◯ Neutral<br>◯ Agree<br>◯ Strongly agree<br>◯ Not applicable
Please indicate briefly the reason(s) for your response to the question above (optional)	________________________
The questions I was asked while working through this case were helpful in enhancing my diagnostic reasoning in this case	◯ Strongly disagree<br>◯ Disagree<br>◯ Neutral<br>◯ Agree<br>◯ Strongly agree<br>◯ Not applicable
Please indicate briefly the reason(s) for your response to the question above (optional)	________________________
The feedback I received was helpful in enhancing my diagnostic reasoning in this case	◯ Strongly disagree<br>◯ Disagree<br>◯ Neutral<br>◯ Agree<br>◯ Strongly agree<br>◯ Not applicable
Please indicate briefly the reason(s) for your response to the question above (optional)	________________________
**Learning effect of consultation**	
After completing this case, I feel better prepared to confirm a diagnosis and exclude differential diagnoses in a real life patient with this complaint	◯ Strongly disagree<br>◯ Disagree<br>◯ Neutral<br>◯ Agree<br>◯ Strongly agree<br>◯ Not applicable
Please indicate briefly the reason(s) for your response to the question above (optional)	________________________

After completing this case, I feel better prepared to care for a real life patient with this complaint	◯ Strongly disagree<br>◯ Disagree<br>◯ Neutral<br>◯ Agree<br>◯ Strongly agree<br>◯ Not applicable
Please indicate briefly the reason(s) for your response to the question above (optional)	______________________
**Overall judgment of case workup**	
Overall, working through this case was a worthwile learning experience	◯ Strongly disagree<br>◯ Disagree<br>◯ Neutral<br>◯ Agree<br>◯ Strongly agree<br>◯ Not applicable
Please indicate briefly the reason(s) for your response to the question above (optional)	______________________
**Open-ended questions**	
Special strenghts of the case	____________________________
Special weaknesses of the case	____________________________
Any additional comments	____________________________

CR: clinical reasoning; VP: virtual patient.

Figure S2. Example of prompt used for the LLM-empowered VP simulation.

Mikael is about to meet his physician for the first time. Mikael is a 68-year-old man who made an appointment a few days back. Apart from the age and sex of the patient, the only information the physician has is that Mikael has sought care because of "ache in the body".

The following is some information about Mikael's condition [shortened]:

- He has always been healthy and has not felt any pain similar to that he is seeking for now.
- Sometimes, he experiences back pain, but it has never been present for that long and it has not had the same character.
- In Mikael's medical charts, it is stated that his blood lipids are above normal.
- Mikael has been advised to eat healthier to avoid developing diabetes and lower his blood lipids.
- Mikael has had a stable blood pressure since he has been on anti-hypertensive treatment.

The following is a dialogue between Mikael and his physician:

- Mikael: Hi doctor.
- Physician: Hello Mikael, my name is Morgan.
- Mikael: Nice to meet you, Morgan. Thanks for seeing me.
- Physician: Of course! Could you describe why you are here today?

Write the next line that Mikael would say.

Figure S3. Interview guide exploring students' perception of virtual patient platforms with regard to acquirement of clinical reasoning skills.

Introduction

- Presentation.
- Purpose of the interview: "explore perceptions and experiences from the usage of virtual patient cases through an AI-driven social robot as compared with a conventional semi-linear computer-based platform, for the training of clinical reasoning skills".
- Tell about the number of interviews and recruitment.
- Contact information, neutrality, and pseudonymity – personal information will be saved on locked servers and coded during analysis. Reported in aggregated level.
- Informed consent form.

Background

- Can you tell me a little bit about your background? Which semester are you attending at the medical programme at this moment and what clinical rotation or course are you attending right now?

Virtual patient cases

- Where were you located when you did the cases? What were your perceptions and thoughts going into this activity? Did you prepare for the activity in any way?
- Did you feel like you got the time that you needed to perform the educational activity? Would you have liked more time or less time?
- How "close" did you feel towards the patient? Did you feel like the physician attending the patient? What are your thoughts regarding the patient encounter?
- What skills did you feel like you got to practice during these virtual patient cases?
- Can virtual patient cases be a complement to meeting real-life patients in the clinic? (quality of the patient encounter)
- Can you please elaborate what the word clinical reasoning means to you?
- Is there any other instance, or anything else you can think of, where virtual patient simulations can be of benefit if implemented? (A test? Examination? Learning through failure? Etc…)

Comparison of the platforms:

- Could you please describe your experience using the AI-driven social robotic platform for virtual patient simulations? What aspects of the platform stood out to you in terms of facilitating clinical reasoning skill development?

- How familiar are you with the conventional semi-linear virtual patient simulation platform? How would you compare your experiences between the two platforms in terms of supporting the practice of clinical reasoning skills?
- In your opinion, what are the main strengths and weaknesses of the AI-driven social robotic platform compared to the conventional semi-linear one for clinical reasoning skill training? How do these differences impact the overall learning experience?
- How do you perceive the level of engagement and immersion offered by each platform during virtual patient simulations? Do you believe one platform provides a more realistic and authentic experience for practicing clinical reasoning skills?
- Can you share any specific instances where the AI-driven social robotic platform positively influenced your ability to develop and apply clinical reasoning skills? How did it differ from your experiences with the conventional semi-linear platform?
- From your perspective, what role does the presence of an AI-driven social robotic platform play in enhancing the learning process for clinical reasoning skills? How does it differ from traditional instructional methods?
- Were there any challenges or limitations you encountered when using the AI-driven social robotic platform for virtual patient simulations? How did these compare to the challenges posed by the conventional semi-linear platform?
- In what ways do you believe the AI-driven platform can be improved to better support the development of clinical reasoning skills? Are there any features or functionalities you would like to see enhanced or added?
- From an instructional standpoint, how do you think educators can leverage the unique aspects of each platform to optimize the learning experience for clinical reasoning skill development?
- Overall, based on your experiences with both platforms, which one do you believe has the most potential to effectively train and improve clinical reasoning skills, and why?
- Overall, do you believe that virtual patient simulations are a good educational tool to practice clinical reasoning skills? Please elaborate.

Conclusion

- Do you have anything to add or that you want to share based on the cases?
- Do you have anything else to add in general?
- Do you have my contact information?
- Thank you for your participation in the project!
