# Supplementary material for: Virtual Patient Simulations Using Social Robotics Combined With Large Language Models for Clinical Reasoning Training in Medical Education: Mixed Methods Study
Source: J Med Internet Res. 2025 Mar 3;27:e63312. doi: 10.2196/63312 (PMC11914843; doi:10.2196/63312)
Supplement: Multimedia Appendix 2 [file jmir_v27i1e63312_app2.pdf]

Table S1. Responses to the clinical reasoning questionnaire within the theme "authenticity of patient encounter".

**Authenticity of patient encounter in the consultation**		
While working on this case, I felt I had to make the same decisions a doctor would make in real life.		
**Response**	**Robot**	**VIC**
Strongly disagree	0 (0.0%)	0 (0.0%)
Disagree	0 (0.0%)	2 (13.3%)
Neutral	1 (6.7%)	0 (0.0%)
Agree	4 (26.7%)	10 (66.7%)
Strongly agree	10 (66.7%)	3 (20%)
Not applicable	0 (0.0%)	0 (0.0%)
While working on this case, I felt I were the doctor caring for this patient.		
**Response**	**Social robot**	**VIC**
Strongly disagree	0 (0.0%)	0 (0.0%)
Disagree	1 (6.7%)	0 (0.0%)
Neutral	1 (6.7%)	2 (13.3%)
Agree	5 (33.3%)	12 (80.0%)
Strongly agree	8 (53.3%)	1 (6.7%)
Not applicable	0 (0.0%)	0 (0.0%)

Data are presented as numbers (percentage).

VIC: virtual interactive case simulator.

Table S2. Responses to the clinical reasoning questionnaire within the theme "professional approach in the consultation".

**Professional approach in the consultation**		
While working through this case, I was actively engaged in gathering the information (e.g., history questions, physical exams, lab tests) I needed, to characterise the patient's problem.		
**Response**	**Social robot**	**VIC**
Strongly disagree	0 (0.0%)	0 (0.0%)
Disagree	0 (0.0%)	0 (0.0%)
Neutral	0 (0.0%)	2 (13.3%)
Agree	3 (20.0%)	4 (26.7%)
Strongly agree	12 (80.0%)	9 (60.0%)
Not applicable	0 (0.0%)	0 (0.0%)
While working through this case, I was actively engaged in revising my initial image of the patient's problem as new information became available.		
**Response**	**Social robot**	**VIC**
Strongly disagree	0 (0.0%)	0 (0.0%)
Disagree	0 (0.0%)	0 (0.0%)
Neutral	1 (6.7%)	1 (6.7%)
Agree	8 (53.3%)	11 (73.3%)
Strongly agree	6 (40.0%)	3 (20.0%)
Not applicable	0 (0.0%)	0 (0.0%)
While working through this case, I was actively engaged in creating a short summary of the patient's problem using medical terms.		
**Response**	**Social robot**	**VIC**
Strongly disagree	0 (0.0%)	0 (0.0%)
Disagree	1 (6.7%)	4 (26.7%)
Neutral	2 (13.3%)	1 (6.7%)
Agree	6 (40.0%)	5 (33.3%)
Strongly agree	6 (40.0%)	5 (33.3%)
Not applicable	0 (0.0%)	0 (0.0%)
While working through this case, I was actively engaged in thinking about which findings supported or refuted each diagnosis in my differential diagnosis.		
**Response**	**Social robot**	**VIC**
Strongly disagree	0 (0.0%)	0 (0.0%)
Disagree	1 (6.7%)	0 (0.0%)
Neutral	3 (20.0%)	1 (6.7%)
Agree	5 (33.3%)	7 (46.7%)
Strongly agree	6 (40.0%)	7 (46.7%)
Not applicable	0 (0.0%)	0 (0.0%)

Data are presented as numbers (percentage).

VIC: virtual interactive case simulator.

Table S3. Responses to the clinical reasoning questionnaire within the theme "coaching during consultation".

Coaching during consultation		
I felt that the case was at the appropriate level of difficulty for my level of training		
**Response**	**Social robot**	**VIC**
Strongly disagree	0 (0.0%)	0 (0.0%)
Disagree	0 (0.0%)	1 (6.7%)
Neutral	0 (0.0%)	2 (13.3%)
Agree	5 (33.3%)	4 (26.7%)
Strongly agree	10 (66.7%)	8 (53.3%)
Not applicable	0 (0.0%)	0 (0.0%)
The questions I was asked while working through this case were helpful in enhancing my diagnostic reasoning in this case		
**Response**	**Robot**	**VIC**
Strongly disagree	0 (0.0%)	0 (0.0%)
Disagree	0 (0.0%)	0 (0.0%)
Neutral	0 (0.0%)	1 (6.7%)
Agree	7 (46.7%)	6 (40.0%)
Strongly agree	7 (46.7%)	6 (40.0%)
Not applicable	1 (6.7%)	2 (13.3%)
The feedback I received was helpful in enhancing my diagnostic reasoning in this case		
**Response**	**Robot**	**VIC**
Strongly disagree	0 (0.0%)	0 (0.0%)
Disagree	0 (0.0%)	1 (6.7%)
Neutral	1 (6.7%)	0 (0.0%)
Agree	5 (33.3%)	6 (40.0%)
Strongly agree	8 (53.3%)	6 (40.0%)
Not applicable	1 (6.7%)	2 (13.3%)

Data are presented as numbers (percentage).

VIC: virtual interactive case simulator.

Table S4. Responses to the clinical reasoning questionnaire within the theme "learning effect of consultation".

Learning effect of consultation		
After completing this case, I feel better prepared to confirm a diagnosis and exclude differential diagnoses in a real life patient with this complaint		
**Response**	**Social robot**	**VIC**
Strongly disagree	0 (0.0%)	0 (0.0%)
Disagree	0 (0.0%)	0 (0.0%)
Neutral	1 (6.7%)	2 (13.3%)
Agree	7 (46.7%)	8 (53.3%)
Strongly agree	7 (46.7%)	5 (33.3%)
Not applicable	0 (0.0%)	0 (0.0%)
After completing this case, I feel better prepared to care for a real life patient with this complaint		
**Response**	**Social robot**	**VIC**
Strongly disagree	0 (0.0%)	0 (0.0%)
Disagree	0 (0.0%)	0 (0.0%)
Neutral	1 (6.7%)	3 (20.0%)
Agree	7 (46.7%)	9 (60.0%)
Strongly agree	7 (46.7%)	3 (20%)
Not applicable	0 (0.0%)	0 (0.0%)

Data are presented as numbers (percentage).

VIC: virtual interactive case simulator.

Table S5. Responses to the clinical reasoning questionnaire within the theme “overall judgment of case workup”.

**Overall judgment of case workup**		
Overall, working through this case was a worthwile learning experience		
**Response**	**Social robot**	**VIC**
Strongly disagree	0 (0.0%)	0 (0.0%)
Disagree	1 (6.7%)	0 (0.0%)
Neutral	0 (0.0%)	0 (0.0%)
Agree	1 (6.7%)	5 (33.3%)
Strongly agree	13 (86.7%)	10 (66.7%)
Not applicable	0 (0.0%)	0 (0.0%)

Data are presented as numbers (percentage).

VIC: virtual interactive case simulator.

Table S6. Responses to the theme "special strengths of the case" in the clinical reasoning questionnaire.

Special strengths of the case	
**Social robot**	**VIC**
1. "Better way of asking questions".	1. "Good revision".
2. "A fun and good alternative to online cases".	2. "Helpful to give structure to thoughts".
3. "Interaction with the patient seemed real".	3. "Very interesting way to learn about a patient case. Way more interactive than the usual case presentations".
4. "Being asked questions by the robot, feeling the 'presence' to take care of the patient, real one-to-one interaction, having to think yourself about what you want to ask".	4. "It is useful to practice real-life conditions, in which the doctor is expected to ask useful questions to the patients and perform certain physical examination procedures. It was also interesting to try and decide what lab exams to ask based on the clinical suspect. Finally, I liked the sense of uncertainty about the case, because diagnosis is never 100% clear in real life, at least at the beginning of medical assessment".
5. "Active thinking about which questions to ask and how to formulate them. Patient asks questions which you have to be able to react to, which makes it harder and more fun".	5. "Teaching case. Very typical presentation and typical lab findings".
6. "Realistic conversation".	6. "Resembles real clinical practice. Conveys the idea that the patient is on your responsibility. The fact that the system should evaluate the quality/quantity of your questions is very challenging".
7. "The flow of the conversation".	7. "The patient does not answer in technical terms which leaves me to interpret the meaning of different symptoms".
8. "Very enjoyable and it was quick to respond".	8. "Very enjoyable I must say, and the interface is nice and super easy to follow and understand. the feedback given is also helpful in helping us understand the case better".
9. "It feels like if you were with a real patient".	9. "Interesting case and good questions".
10. "Well written cases good interaction with the robot that mimics the reality".	10. "The answers of the patient were similar to real life experience".
11. "You can guide your own consultation with your own questions".	11. "Explores all aspects of the consultation".
12. "The patient knows how to answer the questions in a clean way, and gives answers that leads me, as a medical student, to a diagnosis, without giving tricky situations. The clinical picture is clear and simplified".	12. "The fact that it is systematic permits us to be very organised and not forget anything. Also, the lab tests, and the differential diagnosis help a lot in learning and reasoning".
13. "The presence of a robot, talking and instantly responding feels like in real life, pushes you to think quickly and efficiently".	13. "Global and complete view over the patient case with the physical exam and other investigations".
14. "Interactive, open-ended"	14. No response.
15. No response.	15. "Clear complaint, structured workflow".

VIC: virtual interactive case simulator.

Table S7. Responses to the theme "special weaknesses of the case" in the clinical reasoning questionnaire.

**Special weaknesses of the case**	
**Social robot**	**VIC**
1. "Only history no labs etc".<br><br>2. "Listening and talking feels a bit mechanical".<br><br>3. "Not allowing us to pause, making the conversation a bit mechanical".<br><br>4. "Already knowing the diagnosis, the robot doesn't lie/give misleading information (unlike patients...)".<br><br>5. "Doesn't train empathic interactions with patients and social skills".<br><br>6. "Interruptions during your speech".<br><br>7. "It is somewhat mechanical. As true as it can be without a real patient".<br><br>8. "The pauses, not being able to pick up some of my sentences".<br><br>9. No response.<br><br>10. "Lack of expression of emotions".<br><br>11. "Felt more like an exam than a consultation with a patient".<br><br>12. "The robot sometimes repeats previous information by itself when it hears 'ok'".<br><br>13. Not having much time to ask your whole questions/having some information without asking for them.<br><br>14. "Lack of test results/examinations".<br><br>15. No response.	1. "Couldn't ask own questions".<br><br>2. "A bit confusing when you don't know why something is as it is".<br><br>3. "I didn't understand well that I wasn't supposed to ask every question and click on everything".<br><br>4. "It would be even better if the case included videos or pictures, for example for the physical examination part. The students look at the video/pics and assess themselves what they think is wrong in the patient".<br><br>5. "Generally a bit hard to find the right questions in the navigation in my opinion. Takes some time to get used to. Case almost too clear".<br><br>6. "This was my first virtual patient case. In this case, the patient did not expose his main complaints, so I 'asked' a lot of general, unspecific, and even useless questions at the beginning, until I understood the main problems and started focusing on those ones. I would add a just a little broader 'spontaneous' presentation of the patient at the beginning to make it more challenging. However, I would not call it a weakness: the case was very good and very well done".<br><br>7. "Maybe it could be a checklist where you gather information instead of having to open new dialogue interfaces for each question and you get the information in one answer. Especially this applies to the lab work and examination part".<br><br>8. "I was only a little confused as to why there was mention of fever in the 'regarding condition' section. Because there was no mentioning of fever as a presenting complaint in the beginning".<br><br>9. No response.<br><br>10. "The access to all questions and irrelevant examination without getting penalized".<br><br>11. "I believe that this case was maybe 'too' perfect without the limitation of real-life scenarios: limit of time, forget question, angry patient, expensive tests...".<br><br>12. "Not the same feeling of being with a patient as with the robot".<br><br>13. "You don't think too much to get the answers, you don't ask the questions".

	14. No response.<br><br>15. “Fixed questions, could use more feedback”.

VIC: virtual interactive case simulator.

Table S8. Identified themes and sub-themes within the qualitative thematic analysis.

Themes	Sub-themes	Analytical codes
**Realism and immersion**	Authenticity	Feeling like a physician
		Patient presentation
		Responsibility
	Interactivity and engagement	Asking questions in real time
		Level of engagement
		Face projection
**Skill acquisition and development**	CR skills	Importance of CR
		Hypothesis generation
		Knowledge
		Medical history
	Communication skills	Phrasing questions
		Practice communication
		Active communication
	Emotional skills	Empathy
		Emotion
		Personality
**Procedural limitations**	Technical limitations	Interruption
		Information
		Difficulty
	User-related challenges	Stress of interaction
		Nervousness
**Potential for improvement**	NA	Face projection
		Accessibility
		Language

CR: clinical reasoning; NA: not applicable.
